# Lymphocyte integrins mediate entry and dysregulation of T cells by SARS-CoV-2

**DOI:** 10.1038/s41392-023-01348-0

**Published:** 2023-02-27

**Authors:** Mengwen Huang, Xingchao Pan, Xinling Wang, Qingfei Ren, Bei Tong, Xianchi Dong, Gaoxiang Ge, Lu Lu, Shibo Jiang, Jianfeng Chen

**Affiliations:** 1 Key Laboratory of Systems Health Science of Zhejiang Province, School of Life Science, Hangzhou Institute for Advanced Study, University of Chinese Academy of Sciences, Hangzhou, 310024 China; 2 State Key Laboratory of Cell Biology, Center for Excellence in Molecular Cell Science, Shanghai Institute of Biochemistry and Cell Biology, Chinese Academy of Sciences, University of Chinese Academy of Sciences, Shanghai, 200031 China; 3 Key Laboratory of Medical Molecular Virology (MOE/NHC/CAMS), Shanghai Institute of Infectious Disease and Biosecurity, School of Basic Medical Sciences, Shanghai Frontiers Science Center of Pathogenic Microbes and Infection, Fudan University, Shanghai, 200032 China; 4 State Key Laboratory of Pharmaceutical Biotechnology, School of Life Sciences, Nanjing University, Nanjing, 210023 China

**Keywords:** Infection, Lymphocytes

**Dear Editor**,

T-cell infection by SARS-CoV-2 and associated immune responses are correlated with disease severity and prognosis of COVID-19. Lymphopenia is associated with increased disease severity in COVID-19. Significantly lower circulating T and B cell counts were observed in patients who died from COVID-19 compared with survivors. Moreover, SARS-CoV-2 infection causes aberrant lymphocyte activation and dysfunction. COVID-19 patients displayed hyperactivated T cells which are prone to apoptosis and dysregulated cytokine responses.^1^ Some evidence suggests the infection of lymphocytes by SARS-CoV-2, including colocalization of SARS-CoV-2 spike (S) protein and lymphocytes in lung tissues of COVID-19 patients and strands of SARS-CoV-2 sub-genomes in immune cells from bronchoalveolar lavage fluid (BALF) and sputum samples of severe COVID-19 patients.^2^ Angiotensin-converting enzyme 2 (ACE2) and some other reported S protein receptors are barely expressed in lymphocytes. Some recent studies suggest that integrins may act as SARS-CoV-2 receptors, shedding light on the dysregulation of lymphocyte functions by SARS-CoV-2. However, a clear-cut mechanism underlying the crosstalk between SARS-CoV-2 and lymphocyte integrins remains elusive.

Integrins are a family of α/β heterodimeric cell surface adhesion receptors. A panel of integrins is specifically expressed on the surface of lymphocytes, including α4 integrins and β2 integrins. Integrins bind to their ligands by recognizing two types of conserved tripeptide sequences, Arg-Gly-Asp (RGD motif) and Leu/Ile-Asp/Glu-Val/Ser/Thr (LDV motif). The Asp residue forms a critical interaction with the metal ion-dependent site (MIDAS) in integrins.^3^ Of note, the receptor-binding domain (RBD) of SARS-CoV-2 S protein (S-RBD) has three potential integrin-binding motifs: RGD (Arg403-Gly404-Asp405), LDS (Leu441-Asp442-Ser443) and LDI (Leu585-Asp586-Ile587). Moreover, proinflammatory cytokines,^2^ such as IP-10 and SDF-1α, were elevated in the sera of severe patients. These cytokines can induce the activation of integrins on lymphocytes and increase integrin ligand-binding affinity.^3^ These clues suggest that SARS-CoV-2 may use integrins as receptors to enter lymphocytes, thus potentially playing a role in SARS-CoV-2-induced T-cell immune responses.

Analysis of the published single-cell RNA sequencing (scRNA-seq) data of BALFs from patients with severe COVID-19 showed that viral RNAs of SARS-CoV-2 were detected in epithelial cells and multiple immune cells, including, T cells, B cells, plasma cells, natural killer (NK) cells, neutrophils, plasmacytoid dendritic cells (pDCs), myeloid dendritic cells (mDCs), mast cells and macrophages (Supplementary Fig. S1a, b). Notably, T cells harbored a substantial amount of viral RNAs, whereas ACE2 and other reported SARS-CoV-2 receptors were barely detected in T cells (Supplementary Fig. S1c, d). Integrin α4β1, α4β7, αLβ2, and α5β1 were highly expressed in human primary T cells (Fig. 1a). Among the three potential integrin-binding sites in S-RBD (Supplementary Fig. S1e), LDS and LDI were potential binding sites for α4β1, α4β7, and αLβ2 integrins, while RGD was a typical binding motif for α5β1. To investigate whether these integrins can interact with S-RBD, we isolated the membrane fraction of human primary T cells and performed a pull-down assay using S-RBD protein with C-terminal human Fc-tag (S-RBD-hFc) as the bait and integrin subunits as the targets. The results showed interaction between S-RBD and integrin α4, α5, β1, β7, and β2 subunits (Fig. 1b), suggesting an interaction between S-RBD and integrin α4β1, α4β7, αLβ2, and α5β1.

**Fig. 1:**
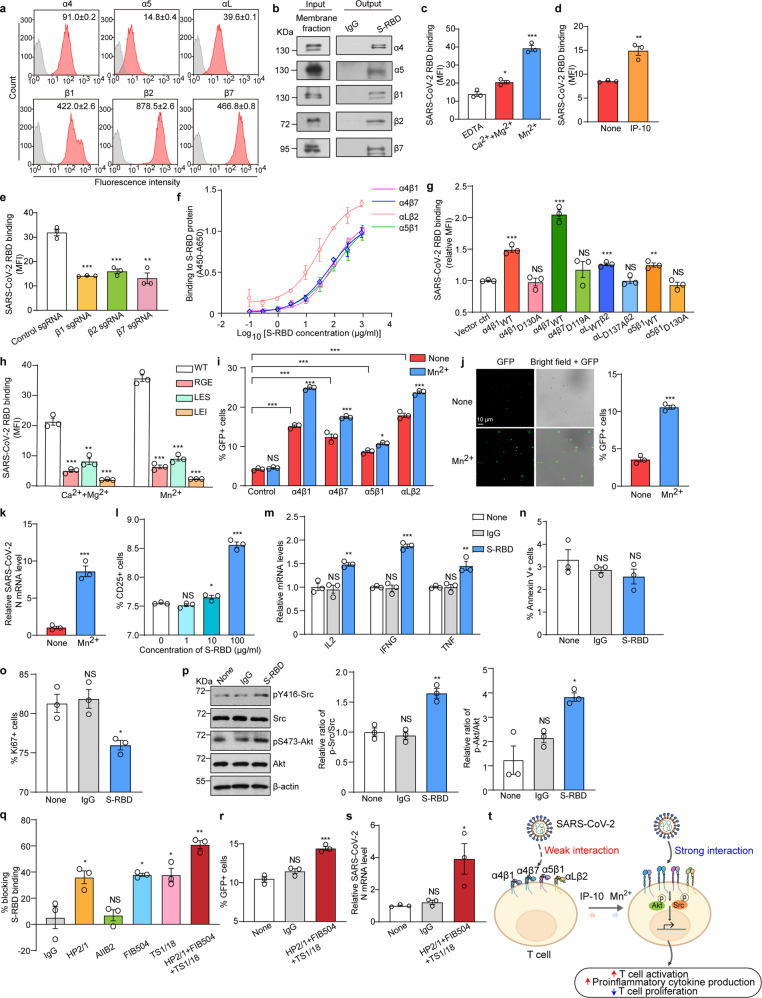
Integrins mediate SARS-CoV-2 T cell entry and dysregulation of T cell response. **a** Expression of integrins on human primary T cells was determined by flow cytometry. Numbers within panels showed the specific mean fluorescence intensities. Gray histogram: mock control. **b** Precipitation of integrins from the membrane fraction of human primary T cells by protein G beads loaded with S-RBD-hFc. The binding was detected by immunoblot. Human IgG was used as isotype control. One representative result of three independent experiments is shown. **c** Binding of soluble S-RBD protein to T cells in different divalent cation conditions. Human primary T cells were incubated with Alexa Fluor 488-labeled S-RBD (100 μg/ml) in the presence of 5 mM EDTA, 1 mM Ca^2+^/Mg^2+^, or 1 mM Mn^2+^, respectively. The binding of soluble S-RBD protein to T cells was examined by flow cytometry and shown as the specific mean fluorescence intensity (MFI) of Alexa Fluor 488-labeled S-RBD (*n* = 3). **d** Binding of soluble S-RBD protein to T cells upon IP-10 treatment. T cells were pretreated with 2 μg/ml IP-10 and then incubated with Alexa Fluor 488-labeled S-RBD (100 μg/ml) in the presence of 1 mM Ca^2+^/Mg^2+^ (*n* = 3). **e** Expression of β1, β2, and β7 integrins was silenced in human primary T cells with the indicated sgRNA, respectively. The binding of soluble S-RBD protein (100 μg/ml) to modified T cells in the presence of 1 mM Mn^2+^ was detected by flow cytometry (*n* = 3). **f** Assessment of the binding affinity of S-RBD protein to immobilized integrin proteins containing headpiece domains (10 μg/ml) in the presence of 1 mM Mn^2+^ using ELISA. (*n* = 3). **g** Binding of soluble S-RBD protein to 293 T cells expressing WT or MIDAS mutant T cell integrins. β1-KO 293 T cells were transfected with WT or MIDAS mutant α4β1, α4β7, αLβ2, and α5β1 integrins. The binding of S-RBD (100 μg/ml) to these cells in the presence of 1 mM Mn^2+^ was measured by flow cytometry (*n* = 3). **h** Effects of integrin-binding motif mutations in S-RBD on the binding of S-RBD protein to T cells. The binding of 100 μg/ml WT or S-RBD mutant (RGE, LES, or LEI) protein to T cells in the presence or absence of 1 mM Mn^2+^ was measured by flow cytometry (*n* = 3). **i** SARS-CoV-2 pseudovirus entry into β1-KO 293 T cells ectopically expressing the indicated T cell integrins. β1-KO 293 T cells expressing the indicated integrins were cocultured with GFP-expressing SARS-CoV-2 pseudovirus in the presence or absence of 0.2 mM Mn^2+^ for 12 h. Supernatants were removed, and the cells were incubated with fresh medium for 36 h. GFP+ cells were quantified by flow cytometry (*n* = 3). **j** SARS-CoV-2 pseudovirus entry into human primary T cells before and after integrin activation. T cells were pretreated with 0.2 mM Mn^2+^ and then incubated with SARS-CoV-2 pseudovirus for 12 h. Supernatants were removed, and the cells were incubated with fresh medium for 36 h. The representative confocal images were shown (left panel). GFP+ cells were quantified by flow cytometry (*n* = 3) (right panel). **k** SARS-CoV-2 authentic virus infection of human primary T cells before and after integrin activation. T cells were pretreated with 0.2 mM Mn^2+^ and then incubated with the SARS-CoV-2 authentic virus for 1.5 h. The supernatants were removed, and the cells were incubated with fresh medium for 24 h. Relative mRNA level of the SARS-CoV-2 N gene was examined in T cells at 24 h post-infection by RT-PCR. **l** Activation of T cells by S-RBD protein treatment. Unactivated human primary T cells were cocultured with 0, 1, 10, or 100 μg/ml S-RBD protein, respectively, for 16 h in the presence of 0.2 mM Mn^2+^. CD25 was stained for evaluation of T cell activation capacity (*n* = 3). **m** Relative mRNA levels of proinflammatory cytokines in T cells upon S-RBD protein treatment. T cells were cocultured with 100 μg/ml S-RBD or human IgG as a control in the presence of 0.2 mM Mn^2+^ for 24 h. Cells were then lysed, and the mRNA levels of the indicated cytokines were measured by RT-PCR (*n* = 3). **n**, **o** Apoptosis and proliferation of T cells upon S-RBD protein stimulation. T cells were cocultured with 100 μg/ml S-RBD or human IgG in the presence of 0.2 mM Mn^2+^ for 30 min. Cells were washed and resuspended in a fresh medium for further incubation at 37 °C for 36 h. The percentage of Annexin V + and Ki67+ cells was examined by flow cytometry (*n* = 3). **p** Immunoblot analysis of phosphorylation of Src (pY416) and Akt (pS473) in T cells upon S-RBD protein stimulation. T cells were cocultured with 100 μg/ml S-RBD or human IgG for 2 h in the presence of 0.2 mM Mn^2+^. One representative result of three independent experiments is shown (left panel). The relative ratios of p-Src/Src and p-Akt/Akt were normalized to the values of the untreated group (None) (*n* = 3) (right panel). **q** The effect of integrin-blocking antibodies on S-RBD binding to T cells. Human primary T cells were pretreated with the indicated antibodies (10 μg/ml of each) followed by incubation with S-RBD protein (100 μg/ml) in the presence of 1 mM Mn^2+^. Mouse IgG was used as isotype control. The blocking efficiency of integrin-blocking antibodies was normalized to the specific MFI of the IgG group (*n* = 3). **r** SARS-CoV-2 pseudovirus entry into T cells in the absence or presence of integrin-blocking antibodies. Human primary T cells were pretreated with mouse IgG or HP2/1 + FIB504 + TS1/18 cocktail (10 μg/ml of each), followed by incubation with GFP-expressing SARS-CoV-2 pseudovirus for 12 h in the presence of 0.2 mM Mn^2+^. GFP + T cells were detected by flow cytometry after 36 h incubation (*n* = 3). **s** SARS-CoV-2 authentic infection of T cells in the absence or presence of integrin-blocking antibodies. Human primary T cells were pretreated with mouse IgG or HP2/1 + FIB504 + TS1/18 cocktail (10 μg/ml of each), followed by incubation with SARS-CoV-2 authentic virus for 1.5 h at 37 °C. Supernatants were removed, and cells were incubated with fresh medium for 48 h. Relative mRNA level of SARS-CoV-2 N gene was examined in T cells at 48 h post-infection by RT-PCR (*n* = 3). **t** The graphical abstract of the study. Data represent the mean ± s.e.m., *p < 0.05, **p < 0.01, ***p < 0.001, NS no significance

Integrin ligand-binding is metal ion-dependent, and its ligand-binding affinity is regulated by divalent cations. Integrins are usually in a low-affinity state in the presence of 1 mM Ca^2+^/Mg^2+^, the physiological concentration of Ca^2+^ and Mg^2+^ in human peripheral blood. The addition of Mn^2+^ can induce strong activation of integrins.^3^ Therefore, we next examined the binding of soluble S-RBD protein to T cells in different divalent cation conditions. In the presence of 1 mM Ca^2+^/Mg^2+^, soluble S-RBD protein showed efficient binding to T cells. S-RBD binding was significantly enhanced by the treatment of T cells with 1 mM Mn^2+^. Removal of divalent cations by EDTA treatment inhibited the binding of S-RBD to T cells (Fig. 1c). Because T cells barely express Fc-receptors for IgG (FcγRs), the Fc tag of S-RBD protein should not affect S-RBD binding to T cells, which was supported by the control experiment showing no binding of human IgG1 to T cells (Supplementary Fig. S2). These data indicate that the binding of S-RBD to T cells is dependent on divalent cations and enhanced upon integrin activation.

Clinical studies have shown that plasma concentrations of proinflammatory chemokines, such as IP-10, were elevated in COVID-19 patients with mild and severe symptoms.^2^ We then assessed the role of IP-10 in regulating S-RBD binding to T cells. Consistent with the fact that chemokines can trigger integrin activation via inside-out signaling,^3^ IP-10 treatment significantly enhanced S-RBD binding to T cells (Fig. 1d).

To confirm the interaction between S-RBD and these integrins, we individually knocked down integrin β1, β2, or β7 in human primary T cells and measured the binding of S-RBD to these modified T cells. Knockdown of integrin β1, β2, or β7 significantly inhibited the binding of S-RBD to T cells (Fig. 1e and Supplementary Fig. S3), suggesting that β1, β2, and β7 integrins all contribute to mediating S-RBD**‒**T cell interaction. Next, we used an enzyme-linked immunosorbent assay (ELISA) to assess the direct protein-protein interaction between S-RBD and the purified integrin proteins. Consistent with the results of soluble S-RBD protein binding to T cells, evident direct interaction was observed between S-RBD and the purified integrin α4β1, α4β7, αLβ2, and α5β1 proteins (Fig. 1f). The half maximal effective concentrations (EC_50_) of S-RBD binding were 83.8 μg/ml for α4β1, 75.8 μg/ml for α4β7, 30.8 μg/ml for αLβ2 and 133.6 μg/ml for α5β1.

MIDAS is the primary ligand-binding site in integrins. For α4β1, α4β7, and α5β1, MIDAS locates in the I domain in β subunit. Integrin αLβ2 has an I domain in the αL subunit, which contains the MIDAS to mediate ligand binding. To explore the role of MIDAS in mediating S-RBD‒integrin binding, we abolished the metal ion binding in MIDAS of β1, β7, and αL subunits by mutating the metal‐coordinating residues to Ala. The wild-type (WT) or mutant α4β1, α4β7, αLβ2, or α5β1 integrin was transiently expressed at a comparable level in β1 knockout (β1-KO) 293 T cell line, which does not express endogenous α4β1, α4β7, αLβ2, and α5β1 integrins (Supplementary Fig. S4). The binding of soluble S-RBD protein to these cells was examined. The results showed that expression of each integrin promoted S-RBD binding in the presence of Mn^2+^, which was fully abolished by MIDAS mutation (Fig. 1g). Thus, MIDAS in α4β1, α4β7, αLβ2, and α5β1 integrins is critical for S-RBD binding.

To investigate the role of the three putative integrin-binding motifs in S-RBD, we mutated the essential integrin-binding residue Asp to Glu in RGD, LDS, and LDI motifs, respectively. The RGE (D405E), LES (D442E), and LEI (D586E) S-RBD mutants were purified (Supplementary Fig. S5a) and the binding of these proteins to human primary T cells was examined. Compared with WT S-RBD, all Glu-substitution mutant S-RBD proteins showed reduced binding to T cells (Fig. 1h), suggesting that the three putative integrin-binding sites contribute to S-RBD–integrin interaction. Among the three mutant S-RBD proteins, the LEI mutant showed the lowest binding to T cells. We tried to purify the RGE, LES, and LEI triple-point S-RBD mutant but failed. The triple-point RBD mutant showed abnormal polymerization (Supplementary Fig. S5b), suggesting an abnormal structure and function of this mutant.

To determine whether SARS-CoV-2 employs integrins to facilitate its T cell entry process, we examined the role of T cell integrins in the entry of 293 T cells by a GFP-expressing lentivirus pseudotyped with SARS-CoV-2 S protein (SARS-CoV-2 pseudovirus). Over-expression of α4β1, α4β7, α5β1 or αLβ2 integrin in β1-KO 293 T cells led to increased GFP signals in the cells, suggesting that these integrins promote SARS-CoV-2 pseudovirus entry. Furthermore, SARS-CoV-2 pseudovirus entry was significantly enhanced by Mn^2+^-induced integrin activation (Fig. 1i and Supplementary Fig. S6a). Consistently, the enhanced entry of human primary T cells by SARS-CoV-2 pseudovirus (Fig. 1j and Supplementary Fig. S6b) and authentic virus (Fig. 1k) was observed upon integrin activation by Mn^2+^ treatment. Collectively, these results indicate that T cell integrins mediate SARS-CoV-2 cell entry, which can be enhanced by integrin activation.

Most COVID-19 patients exhibited lymphopenia, lymphocyte hyperactivation, and high proinflammatory cytokine levels.^1^ We next investigated T cell responses upon stimulation with S-RBD protein. Notably, S-RBD induced the activation of human primary T cells in a dose-dependent manner (Fig. 1l). Meanwhile, mRNA expression levels of proinflammatory cytokines, such as IL-2, IFN-γ, and TNF-α, were elevated in T cells (Fig. 1m). Although the apoptosis of T cells remained unchanged upon S-RBD treatment (Fig. 1n), the reduced Ki67 signals in S-RBD treated cells suggested the suppressed T cell proliferation, which may be one potential reason for lymphopenia in COVID-19 patients (Fig. 1o).

The binding of ligands to integrin can trigger the activation of intracellular signaling pathways. Stronger phosphorylation of Src and Akt were observed in T cells upon stimulation with S-RBD protein, suggesting the involvement of Src and Akt signaling pathways in S-RBD‒induced T cell responses (Fig. 1p). These S-RBD‒induced integrin downstream signaling pathways are related to proinflammatory cytokine production and cell survival,^4^ which may be triggered by SARS-CoV-2 engagement.

Next, we investigated the effects of four blocking antibodies for integrin α4, β1, β2, and β7 on S-RBD binding to T cells (Fig. 1q). Integrin α4 blocking antibody HP2/1, integrin β7 blocking antibody FIB504 and integrin β2 blocking antibody TS1/18 significantly inhibited the binding of S-RBD to T cells. Integrin β1 blocking antibody AIIB2 showed no inhibition. Combined treatment with HP2/1, FIB504, and TS1/18 exhibited the greatest inhibition of S-RBD binding to T cells.

In contrast to the inhibitory effect on S-RBD binding to T cells, the combined treatment with HP2/1, FIB504 and TS1/18 enhanced the entry of T cells by SARS-CoV-2 pseudovirus (Fig. 1r) and authentic virus (Fig. 1s). Since integrins are highly dynamic on the plasma membrane, many routes can trigger integrin clustering, internalization, and endocytosis.^5^ We hypothesized that these integrin-blocking antibodies might induce integrin clustering and internalization, facilitating SARS-CoV-2 entry into T cells. Thus, the usage of integrin-blocking antibodies to inhibit SARS-CoV-2 entry needs further careful investigation. The development of blocking antibodies targeting S-RBD instead of integrin to abolish S-RBD‒integrin interaction may be an alternative approach to preventing SARS-CoV-2 entry into T cells.

In this study, we identified and characterized α4β1, α4β7, αLβ2, and α5β1 integrins as SARS-CoV-2 receptors on T cells, which bound to S-RBD and synergistically facilitated the entry of SARS-CoV-2 into T cells, followed by dysregulation of T cell functions. Integrin activation by proinflammatory chemokines such as IP-10 promoted S-RBD binding, thus enhancing SARS-CoV-2 entry into T cells (Fig. 1t).

In addition to T cells, B cells, plasma cells, NK cells, and neutrophils barely express the known SARS-CoV-2 receptors (Supplementary Fig. S1c). It is noteworthy that these immune cells express integrins, including but not limited to α4β1, α4β7, αLβ2, and α5β1, suggesting that integrins may act as major receptors for SARS-CoV-2 to infect these immune cells. For example, α4β1 and α4β7 integrins are highly expressed in B cells and plasma cells, which may promote SARS-CoV-2 entry into and dysregulation of these cells. This might provide some clues for the lower circulating antibody titer in some COVID-19 patients.

RGD-binding integrin α5β1, the major receptor for fibronectin, is enriched in epithelial cells of many tissues.^5^ Our data suggest that α5β1 may bind to SARS-CoV-2 S-RBD. Of note, SARS-CoV-2 infection was observed in many extra-pulmonary tissues, such as brain tissue, cardiovascular tissue, and lymphoid tissue, where ACE2 expression was very low.^2^ Therefore, it is likely that cells are infected via α5β1**‒**SARS-CoV-2 interaction across these organs. Besides α5β1, αVβ3, αVβ5, αVβ6, αVβ8, α8β1, and αIIbβ3 integrins can also bind to RGD motif. These RGD-binding integrins are widely expressed in different tissues, which might also contribute to SARS-CoV-2 entry.

Lymphopenia and cytokine storm are associated with disease severity in most COVID-19 patients. Our data showed that the binding of S-RBD to integrins on T cells not only suppressed T cell proliferation but also promoted T cell activation and proinflammatory cytokine secretion. The dysregulation of T cells by S-RBD binding may therefore result in aberrant T cell immune responses and increase the severity of COVID-19.

In summary, our study demonstrates that integrins act as SARS-CoV-2 receptors on T cells and mediate entry and dysregulation of T cells by SARS-CoV-2. Blocking S-RBD**‒**integrin interaction may be a strategy for preventing SARS-CoV-2 entry into T cells and associated immune dysregulation in COVID-19 patients.

## Supplementary information

Supplementary Materials-SIGTRANS-08782R

## Data Availability

The data used and analyzed in this study are available in the main text and the Supplementary Materials. Any other raw data that support the findings of this study are available from the corresponding author upon reasonable request.
