# Supplementary material for: Lymphocyte integrins mediate entry and dysregulation of T cells by SARS-CoV-2
Source: Signal Transduct Target Ther. 2023 Feb 27;8:84. doi: 10.1038/s41392-023-01348-0 (PMC9969015; doi:10.1038/s41392-023-01348-0)
Supplement: Supplementary file 1 — Supplementary Materials-SIGTRANS-08782R [file 41392_2023_1348_MOESM1_ESM.docx]

Supplementary Materials for

**Lymphocyte integrins mediate entry and dysregulation of T cells by SARS-CoV-2**

Mengwen Huang^1,2#^, Xingchao Pan^2#^, Xinling Wang^3#^, Qingfei Ren^2^, Bei Tong^4^, Xianchi Dong^4^, Gaoxiang Ge^1,2^, Lu Lu^3^, Shibo Jiang^3*^, Jianfeng Chen^1,2*^

^1^ Key Laboratory of Systems Health Science of Zhejiang Province, School of Life Science, Hangzhou Institute for Advanced Study, University of Chinese Academy of Sciences, Hangzhou 310024, China

^2^ State Key Laboratory of Cell Biology, Center for Excellence in Molecular Cell Science, Shanghai Institute of Biochemistry and Cell Biology, Chinese Academy of Sciences, University of Chinese Academy of Sciences, Shanghai 200031, China

^3^ Key Laboratory of Medical Molecular Virology (MOE/NHC/CAMS), Shanghai Institute of Infectious Disease and Biosecurity, School of Basic Medical Sciences, Shanghai Frontiers Science Center of Pathogenic Microbes and Infection, Fudan University, Shanghai 200032, China

^4^ State Key Laboratory of Pharmaceutical Biotechnology, School of Life Sciences, Nanjing University, Nanjing 210023, China

^#^ These authors contributed equally to this work

^*^ Corresponding authors

Correspondence to: jfchen@sibcb.ac.cn (J.C.); shibojiang@fudan.edu.cn (S.J.)

**This PDF file includes:**

Materials and Methods

Supplementary Fig. S1 to S6

**Materials and Methods**

*Cell lines*

293T cells were grown in DMEM medium supplemented with 1 % L-glutamine, 10 % FBS and 1 % penicillin-streptomycin.

*Human primary T cell culture*

Human peripheral blood mononuclear cells (PBMCs) were isolated from peripheral blood of healthy donors by density gradient centrifugation with the Lymphoprep reagent (STEMCELL). PBMCs were used for human T cell separation by Human T Cell Isolation Kit (STEMCELL). Human T cells were resuspended at 1×10^6^ cells/ml in RPMI 1640 medium supplemented with 10 % FBS, 1 % L-glutamine, 1 % penicillin-streptomycin, 10 ng/ml human IL-2, and stimulated with the Human CD3/CD28 T Cell Activator (STEMCELL) for 3 days.

*Antibodies and reagents*

Antibodies for flow cytometry: Anti-human integrin α4 antibody (9F10), Anti-human integrin β1 antibody (AIIB2), Anti-human integrin β7 antibody (FIB504) were prepared with hybridomas (Developmental Studies Hybridoma Bank). Anti-human integrin α5 antibody (NKI-SAM-1) and Anti-human integrin β2 antibody (TS1/18) were from BioLegend. Alexa Fluor 647 goat anti-mouse, goat anti-rat IgG (H+L) polyclonal antibodies were from Invitrogen. Antibodies for immunoblot analysis: Anti-human integrin α4 antibody (EPR1355Y), Anti-human integrin α5 antibody (EPR7854), Anti-human integrin β1 antibody (EP1041Y), Anti-human integrin β7 antibody (EPR1357), and Anti-human integrin β2 polyclonal antibody were from Abcam. Anti-Akt antibody (11E7), Anti-phosphorylated Akt at Ser473 antibody (193H12), Anti-Src antibody (36D10), Anti-phosphorylated Src at Tyr416 polyclonal antibody were from Cell Signaling Technology. HRP anti-β-actin (6G3) was from Multi Sciences. HRP anti-human IgG (H+L) antibody was from Abcam.

TMB substrate solution was from Beyotime. Alexa Fluor 488 Protein Labeling Kit was from Invitrogen.

*Protein expression and purification*

To express S-RBD of SARS-CoV-2, S-RBD (residues R319-S591) was cloned into vector pcDNA3.4. Human IgG1 Fc tag was cloned downstream of S-RBD. RGE (D405E), LES (D442E) and LEI (D586E) S-RBD mutants were constructed by Quickchange (Agilent Technologies). Recombinant S-RBD protein fused with human IgG1 Fc was expressed in 293T cells, and isolated by affinity chromatography using Protein A Agarose (Thermo Fisher Scientific). For the expression of headpieces of integrins, headpieces of α or β subunit were cloned into pcDNA3.1 or pEF1 vector, respectively. 6×His tag or strep tag II was cloned downstream of headpiece of α or β subunit separately. Headpieces of the indicated integrins were expressed in 293T cells and purified by affinity chromatography using Ni^2+^-charged resins (QIAGEN) and Strep-Tactin resins (IBA).

*Soluble S-RBD binding assay*

Soluble S-RBD binding assay was performed as described ^1^. Cells were washed twice in HBS (20 mM Hepes, 150 mM NaCl) containing 1 mM Ca^2+^/Mg^2+^ or 1 mM Mn^2+^, and 100 μg/ml Alexa Fluor 488-labelled S-RBD protein was added to cell suspension and incubated for 30 min at 4 ℃. Cells were washed twice and detected with a FACS Celesta (BD Biosciences). Data were analyzed using FlowJo software.

*Pseudotyped SARS-CoV-2 infection assay*

A GFP-expressing SARS-CoV-2 pseudovirus was packaged and used to infect cells as previously described ^2^. Briefly, 293T cells were incubated with DMEM supplemented with 10% FBS, penicillin-streptomycin and L-glutamine. The next day, cells were cotransfected with pLenti-GFP, psPAX2 and SARS-CoV-2 S plasmids at mass ratio 3:1:1. Supernatants were collected at 72 h post transfection, centrifuged at 4000 g for 5 min, and passed through a 0.45 μm filter, followed by concentration using CP100NX Ultracentrifuge (Hitachi). GFP-expressing pseudoviruses were titrated by Lenti-Pac HIV RT-PCR Titration Kits (GeneCopoeia) according to the manufacturer’s protocol and stored at -80 ℃. Activated T cells were infected with the pseudovirus (5×10^5^ RNA copies) by centrifugation at 1500 g for 1.5 h at 30 ℃. After 12 h incubation with pseudovirus at 37 ℃, cells were resuspended in fresh media, followed by an additional 36 h incubation before analysis by flow cytometry to determine GFP expression.

*Authentic SARS-CoV-2 infection of human primary T cells*

The authentic virus infection assay was performed according to the previous study ^3,4^. T cells were infected with SARS-CoV-2 (nCoV-SH01, GenBank number: MT121215.1) at 1 TCID50 per cell. After incubation at 37 ℃, 5% CO_2_ for 1.5 h, T cells were centrifuged at 850 g for 7 min, at 4 ℃ to remove supernatants. Then cells were suspended with medium (RPMI 1640 medium supplemented 10% FBS, 1 % L-glutamine, 1 % penicillin-streptomycin, 10 ng/ml human IL-2) and seeded into wells of 96-well plate for an additional 24 h. The T cells were collected by centrifugation at 850 g for 7 min, at 4 ℃. Trizol reagent (Takara) was used to extract RNA in T cells. RT-PCR assay was run with One Step TB Green® PrimeScript™ RT-PCR Kit II to quantify viral RNA load. The sequences of primers and probes are as follows:

SARS-CoV-2 Nucleocapsid (N) gene-F: 5’- GGGGAACTTCTCCTGCTAGAAT -3’,

SARS-CoV-2 N gene R: 5’- CAGACATTTTGCTCTCAAGCTG -3’;

GAPDH-F: 5’- GGGCTCTCCAGAACATCATCC -3’,

GAPDH-R: 5’- GTCCACCACTGACACGTTGG -3’.

*ELISA*

Wells of a 96-well plates were coated with 10 μg/ml integrin headpiece at 4℃ overnight. The wells were washed with HBS three times and blocked using 2% BSA in HBS for 1 h at 37 ℃. The wells were incubated with S-RBD-hFc protein in HBS buffer containing 1 mM Mn^2+^ for 4 h at 37 ℃. Following three times washes with HBST (HBS + 0.1 % Tween-20 + 1 mM Mn^2+^), HRP conjugated anti-human IgG (H+L) antibody was added and incubated at 37 ℃ for 1 h. Wells were washed three times with HBST and TMB substrate solution was added to each well. While protected from light, wells were incubated at room temperature for 2-5 min. 1M H_2_SO_4_ was added to stop reaction. Absorbance was measured at 450 nm (A450) and 650 nm (A650).

*Activation, proliferation and apoptosis*

After washed with PBS, 2x10^5^ human primary T cells were resuspended in medium (RPMI 1640 medium supplemented 10% FBS, 1 % L-glutamine, 1 % penicillin-streptomycin, 10 ng/ml human IL-2 and 0.2 mM Mn^2+^) containing 100 μg/ml human IgG or S-RBD, followed by incubation at 37 ℃ for 16 h. Then the percentage of activated T cells was detected by FACS Celesta (BD Biosciences).

For T cell proliferation, after incubation in the indicated conditions for 30 min, the medium was removed by centrifugation at 850 g, 4 ℃ for 7 min. Cells were resuspended in fresh medium and seeded in a 48-well plate. After 36 h incubation, cells were collected for further detection. Cells were washed in cold PBS followed by fixation and permeabilization using Foxp3/Transcription Factor Staining buffer set (Invitrogen). Cells were then stained using Alexa Fluor 700 anti-Ki67 (Invitrogen). Cells were washed, resuspended in PBS and then detected by Beckman cytoflex LX, and data were analyzed by FlowJo software.

For T cell apoptosis, cells were washed in cold PBS and stained for Annexin V and PI using Annexin V-FITC/PI Apoptosis Detection Kit (Vazyme) according to the manufacturer’s instructions. Cells were detected by Beckman cytoflex LX, and data were analyzed by FlowJo software.

*RNA isolation and real-time quantitative PCR*

Total RNA was extracted from cells with TRIzol reagent according to the manufacturer’s instructions (Invitrogen). For cDNA synthesis, RNA was reverse-transcribed with an M-MLV reverse transcriptase (Promega). Then cDNA was amplified by real-time PCR with a SYBR Premix ExTaq Kit (TaKaRa) on a QuantStudio 6 Flex Real-Time PCR System (Applied Biosystems). The expression of target genes was normalized to the expression of housekeeping gene GAPDH. Primers:

GAPDH-F: 5’- CCTGTTGCTGTAGCCGTATTCA -3’,

GAPDH-R: 5’- CCAGGTTGTCTCCTGCGACTT -3’;

TNF-F: 5’- CTCTTCTGCCTGCTGCACTTTG -3’,

TNF-R: 5’- ATGGGCTACAGGCTTGTCACTC -3’;

IFNG-F: 5’- TCGGTAACTGACTTGAATGTCCA -3’,

IFNG-R: 5’- TCGCTTCCCTGTTTTAGCTGC -3’;

IL2-F: 5’- TCCTGTCTTGCATTGCACTAAG -3’,

IL2-R: 5’- CATCCTGGTGAGTTTGGGATTC -3’.

*Silencing of integrin β1, β2, β7 in human primary T cells*

For integrin silencing experiments, sgRNAs were cloned into the vector lenticrispr v2 (Addgene). The 20-nucleotide sgRNA sequence was 5’-GGATTTTCTATGTCATCTGG-3’ for the ITGB1 gene, 5’-GCCCCGACTCGATGCATTCC-3’ for the ITGB2 gene, and 5’-GCGGCGCTGCGCCCGACGAG-3’ for the ITGB7 gene. β1-KO 293T cell line was generated using sgRNA 5’-GGATTTTCTATGTCATCTGG-3’. The sgRNA-resistant synonymous mutation was introduced into integrin β1 construct using QuickChange (Agilent Technologies) for re-expression of these integrins in the β1-KO 293T cell line. sgRNAs lentivirus were generated by cotransfecting plasmids encoding control sgRNA or targeting sgRNAs, psPAX2, and pMD2.G at 5:3:2 mass ratio into 293T cells. Supernatants were collected at 72 h post transfection, centrifuged at 4000 g for 5 min, then passed through a 0.45 μm filter, followed by concentration using CP100NX Ultracentrifuge (Hitachi) and stored at -80 ℃. T cells were infected with the lentivirus by centrifugation at 1500 g for 1.5 h at 30 ℃. After 12 h incubation with lentivirus at 37 ℃, cells were resuspended in fresh media, followed by an additional 36 h incubation before analysis by immunoblot to measure knockout efficiency.

*Pull-down assay*

Human IgG1 Fc-tagged S-RBD protein bound to protein G resins (Millipore) was incubated with membrane fraction of human primary T cells for 2 h at 4 ℃, and bound proteins were detected with the indicated integrins antibodies by immunoblot.

*Immunoblot assay*

Cells were washed and lysed with lysis buffer (20 mM Hepes, pH 7.4, 150 mM NaCl, 1 % Triton X-100, 0.05 % Tween-20, Complete protease inhibitor cocktail tablets and PhosSTOP phosphatase inhibitor cocktail tablets) on ice for 30 minutes. Cell lysates were then analyzed by immunoblot for Src, pY416-Src, Akt, pS473-Akt, and β-actin. β-actin was detected by immunoblot as a loading control. Intensity analyses were conducted using Image J software.

*Analysis of the COVID-19 BALF dataset*

Expression of the indicated genes expression in cells from human tissues were analyzed using a recently published single cell RNA-seq data collected from the GEO database through the accession number GSE145926. Seurat (version 4.1.3) was then used for downstream analyses. Integration analysis of the datasets from patients was performed with default parameters. The following criteria were then applied to filter all the cells: gene number between 200 and 6000 and mitochondrial gene percentage < 10 %. After filtering, we selected the 2000 most highly variable genes by default and scaled all genes (z-scores). Principal component analysis (PCA) was performed on the gene expression of these 2000 genes. Harmony (v.0.1.0) batch correction was performed to remove multi-dataset-specific effects. The first 10 principal components were used for cell clustering. Then UMAP was performed on the matrix for visualization. To visualize the data, the Seurat functions “DimPlot”, “FeaturePlot”, and “DotPlot” were used.

*Statistical Analysis*

Statistical significance was determined by two-tailed Student’s *t* test or two-way ANOVA (GraphPad, version 8.4.3). The resulting p values are indicated as follows: NS, no significance; *, p < 0.05; **, p < 0.01; ***, p < 0.001. Data represent the mean ± S.E.M. of at least three independent experiments.

**References**

1 Rose, D. M., Cardarelli, P. M., Cobb, R. R. & Ginsberg, M. H. Soluble VCAM-1 binding to alpha 4 integrins is cell-type specific and activation dependent and is disrupted during apoptosis in T cells. *Blood.* **95**, 602-609, (2000).

2 Wang, S. et al. AXL is a candidate receptor for SARS-CoV-2 that promotes infection of pulmonary and bronchial epithelial cells. *Cell Res*. **31**, 126-140, (2021).

3 Guo, L. et al. Engineered trimeric ACE2 binds viral spike protein and locks it in "Three-up" conformation to potently inhibit SARS-CoV-2 infection. *Cell Res.* **31**, 98-100, (2021).

4 Shen, X. R. et al. ACE2-independent infection of T lymphocytes by SARS-CoV-2. *Signal Transduct Target Ther*. **7**, 83, (2022).


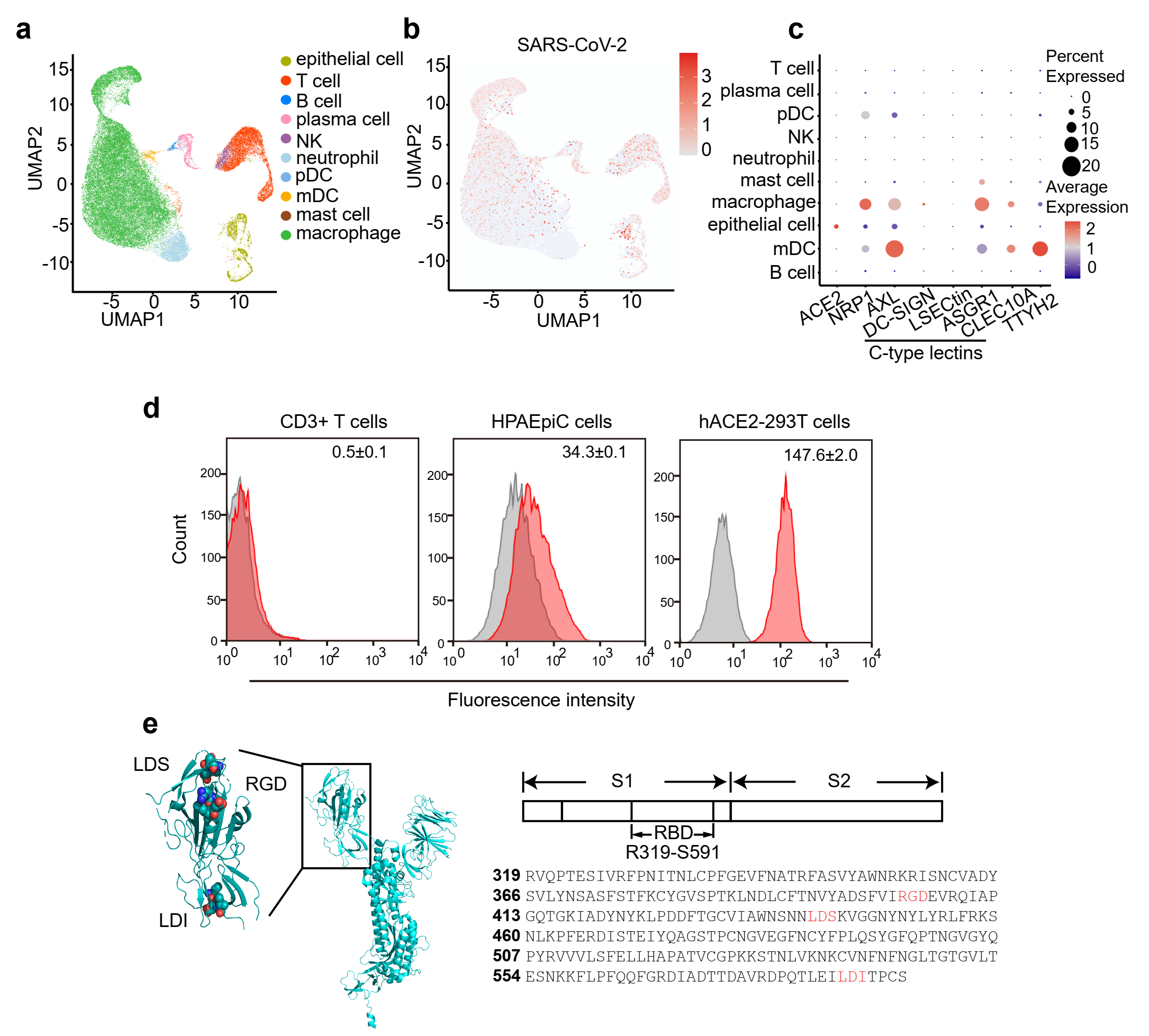


**Supplementary Fig. S1. The binding potential of SARS-CoV-2 S-RBD to T cells**

**a, b** Uniform manifold approximation and projection (UMAP) visualizations of single cells isolated from BALF from nine individuals with mild and severe COVID-19, with coloured clusters labelled by manual cell type annotation. Levels of SARS-CoV-2 viral RNA were plotted.

**c** Expression levels of the reported receptors for SARS-CoV-2 in multiple cell types.

**d** Flow cytometry analysis of human primary T cells, HPAEpiC cells and hACE2-293T cells stained for surface level of ACE2. Numbers within the panel showed the specific mean fluorescence intensities of Polyclonal antibody against ACE2. Grey histogram: mock control.

Data represent the mean ± s.e.m. (*n* = 3).

**e** Diagram of the three potential integrin-binding motifs in SARS-CoV-2 S-RBD. Illustration (left panel) of the three potential integrin-binding motifs in the crystal structure of SARS-CoV-2 spike protein (PDB: 6VXX). Zoomed area: RBD. RGD, LDS and LDI motifs are highlighted in red (right panel).


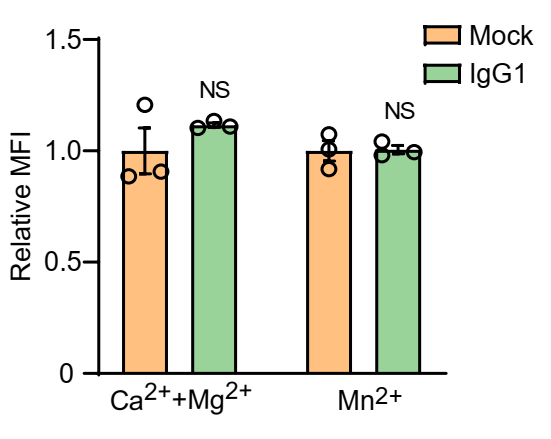


**Supplementary Fig. S2. Binding of soluble human IgG1 isotype to T cells in different divalent cation conditions**

Human primary T cells were incubated with Alexa Fluor 488-labelled human IgG1 isotype (100 μg/ml) in the presence of 1 mM Ca^2+^/Mg^2+^ or 1 mM Mn^2+^, respectively. Untreated T cells were used as mock. The binding was examined by flow cytometry, and the specific mean fluorescence intensity (MFI) of Alexa Fluor 488-labelled human IgG1 isotype was normalized to the mock group.

Data represent the mean ± s.e.m., NS, no significance (two-tailed Student’s *t* test).


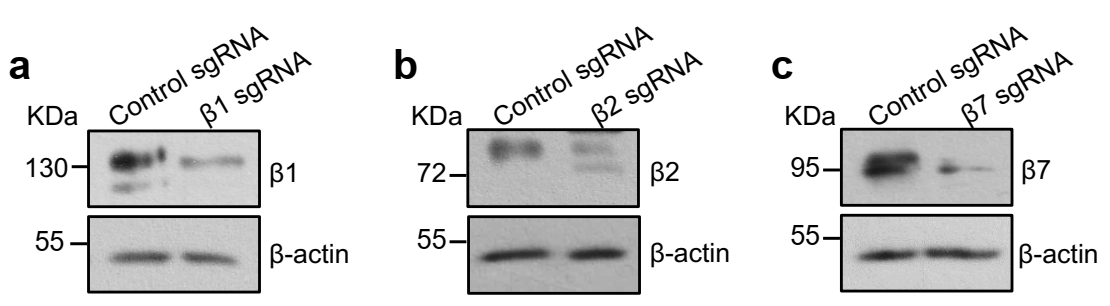


**Supplementary Fig. S3. Silencing efficiency of β1, β2 and β7 integrins in T cells**

**a-c** Expression of β1, β2 and β7 integrins was silenced in human primary T cells with the indicated sgRNA, respectively. Immunoblot analysis of integrin β1, β2 and β7 in cell lysates. One representative result of three independent experiments is shown.


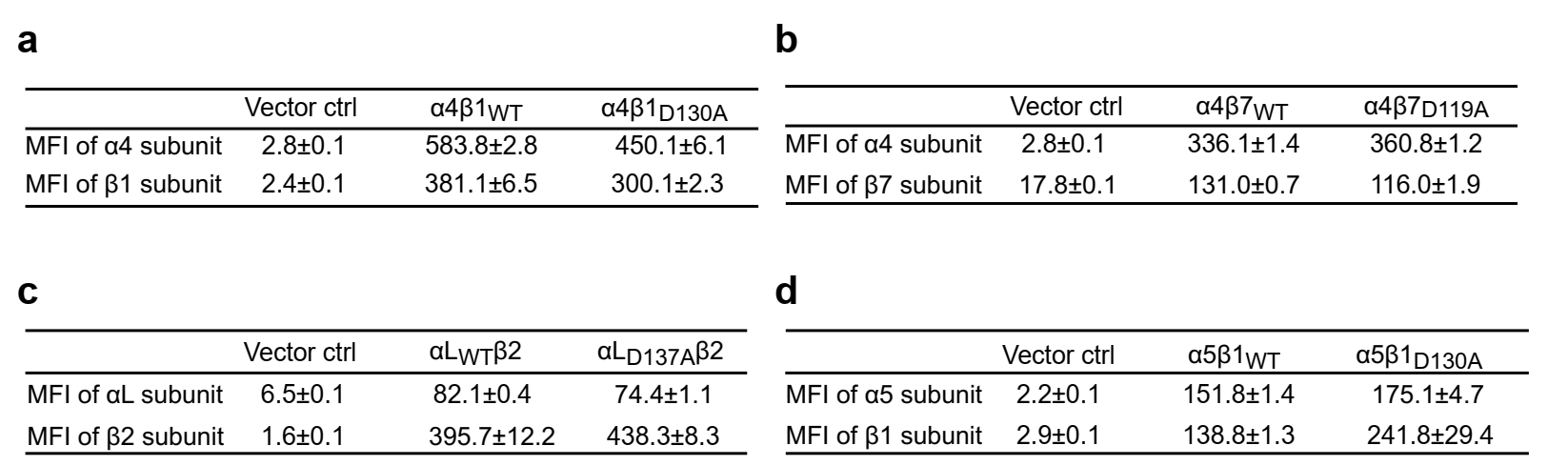


**Supplementary Fig. S4. Expression of the indicated integrins on β1-KO 293T cells**

**a-d** Cell surface expression of integrin α4β1 (**a**), α4β7 (**b**), αLβ2 (**c**) or α5β1 (**d**) was determined by flow cytometry. Numbers within the table showed the specific mean fluorescence intensities.

Data represent the mean ± s.e.m. (*n* = 3).


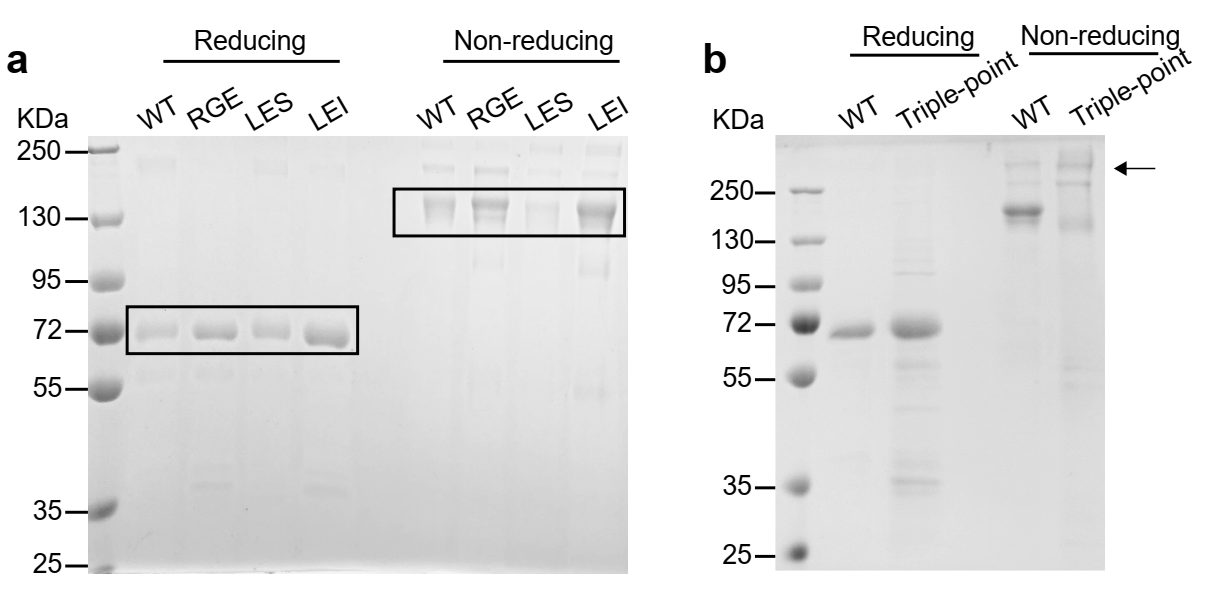


**Supplementary Fig. S5. SDS-PAGE analysis of the purified S-RBD proteins**

**a,b** SDS-PAGE analysis of WT or S-RBD mutants. Framed areas in (**a)** represent target bands of S-RBD proteins. Arrow in (**b**) indicates the abnormal polymerization band of triple-point S-RBD mutant (RGE+LES+LEI).


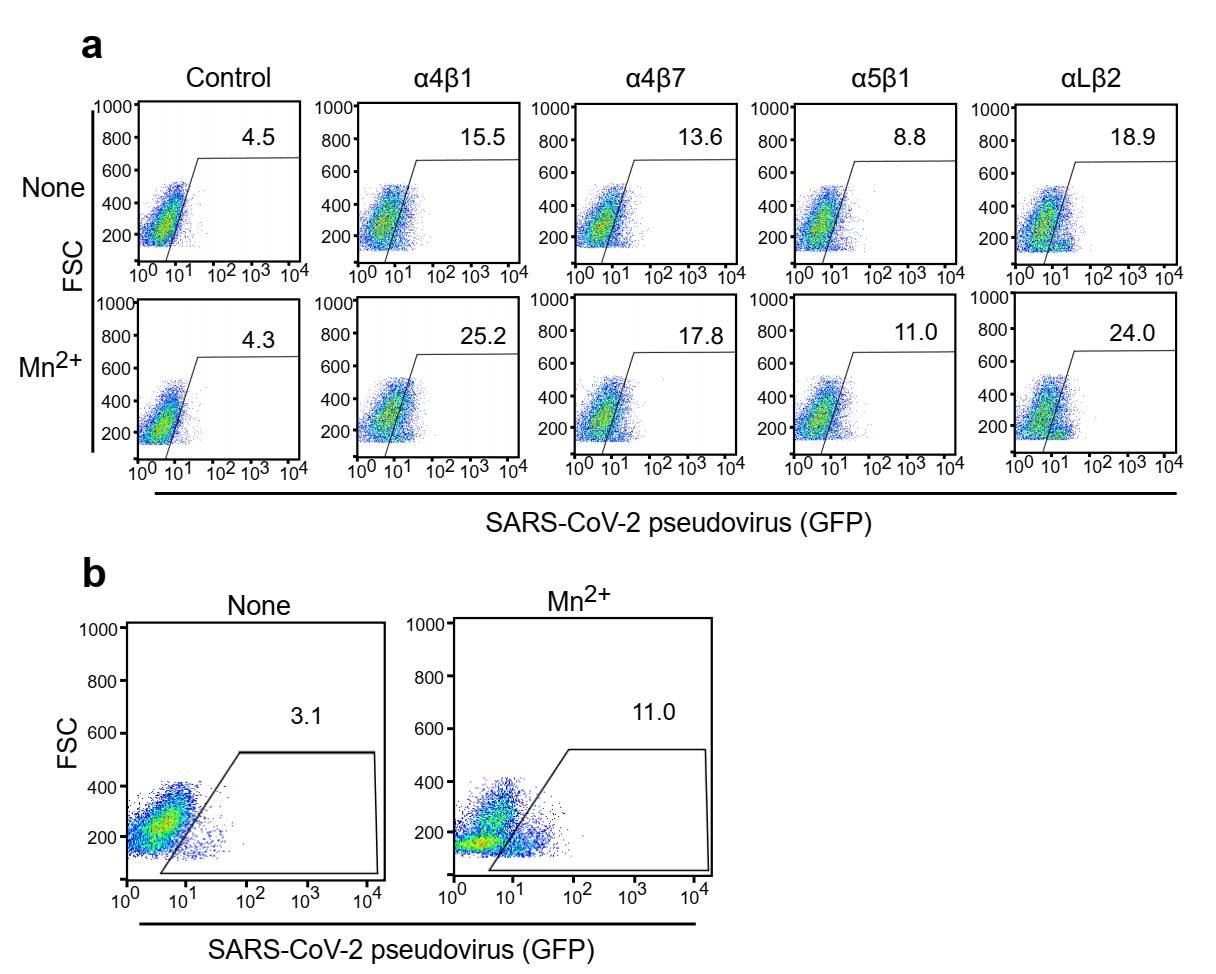


**Supplementary Fig. S6. Integrins mediate SARS-CoV-2 infection of T cells**

**a** Representative flow cytometry plots for SARS-CoV-2 pseudovirus infection of β1-KO 293T cells ectopically expressing the indicated T cell integrins.

**b** Representative flow cytometry plots for SARS-CoV-2 pseudovirus infection of human primary T cells before and after integrin activation.
